# Ethical perspectives of fertility preservation through experts’ opinions

**DOI:** 10.3389/frph.2026.1952541

**Published:** 2026-09-08

**Authors:** Silviya Aleksandrova-Yankulovska, Marcin Orzechowski, Katharina Hancke, Karin Bundschu, Florian Steger

**Affiliations:** 1 Institute of the History, Philosophy and Ethics of Medicine, Ulm University, Ulm, Germany; 2 Department of Gynaecology and Obstetrics, Ulm University, Ulm, Germany

**Keywords:** ethics, experts, fertility preservation, interviews, thematic analysis

## Abstract

**Background:**

Fertility preservation is the process of saving or protecting a person's reproductive substance of human origin intended to be used later in that person's life. Fertility preservation technologies have benefits and risks. Among the most discussed issues is the access to these technologies and their funding. Our study aims at the identification and analysis of specific ethical challenges associated with fertility preservation through the perspective of experts in this field.

**Methods:**

Between March and July 2026, 16 qualitative interviews with experts were conducted. The interview guide consisted of 12 open-ended main questions and 5 follow-up questions. The content of the interviews was analysed using thematic analysis by Braun and Clarke.

**Results:**

Themes identified through the method of thematic analysis were: 1) autonomous decision-making; 2) benefits and risks of fertility preservation; 3) access to fertility preservation; and 4) funding. Within the themes, sub-themes could be identified. The experts supported the involvement of children in decision-making in an age-appropriate manner. Most experts did not support the allowance of postmortem reproduction, but supported information provision in this regard. Medical benefits and risks prevailed over social benefits and risks. All patients’ groups, including those for non-medical indications, should have access to fertility preservation, but funding through health insurance was unanimously supported only for medical indications.

**Conclusion:**

The decisions about fertility preservation are decisions influencing the rest of the life of the patients and the fulfilment of the right to reproduction. All patient groups that can benefit from fertility preservation technologies should have access to them, including minors, healthy women, and transgender individuals. Medical indications for fertility preservation should be covered by health insurance, while the funding of non-medical indications is still debatable. The importance of informed consent for fertility preservation was underlined, particularly in paediatric cancer cases, where the involvement of children in decision-making is supported in accordance with their maturity. As successful as fertility preservation technologies might be, full, proper, and individualised information should be provided to patients to avoid the risk of false hope.

## 1 Introduction

Fertility preservation is “the process of saving or protecting a person's reproductive substance of human origin intended to be used later in that person's life” (1).

Fertility preservation technologies were developed to support cancer patients in their wish for genetically related offspring (2, 3). Currently, other user groups have shown interest in these technologies. Among them, the most studied are healthy women opting for social egg freezing and transgender individuals (4–8). Social egg freezing is the cryopreservation of unfertilised oocytes for non-medical reasons to preserve a woman's future reproductive potential. Social egg freezing is chosen by women who wish to delay childbearing because of personal, social, or career reasons. The aim is to preserve eggs at a younger age, when their quality is generally higher, for possible future use in assisted reproduction (9).

Recognised benefits of fertility preservation include: reduced risk of miscarriage and chromosomal abnormalities in women with social egg freezing (10), sense of security (11) and feeling of relief (12), better preparedness for parenthood (10), opportunity to stay longer and perform better in the labor market (13), to name a few. On the other side, fertility preservation is related to risks, such as delay in treatment of cancer patients and transgender individuals (14, 15). The delay in treatment affects more women than men due to the more complicated procedure of obtaining oocytes after ovarian stimulation (10). Other risks that affect women are ovarian hyperstimulation syndrome (10) and laparoscopy complications (16). Additional risks for all patients include reseeding the cancer when transplanting reproductive tissue (11), risk of misinformation (13, 17), social and emotional risks in children born after social egg freezing and having older parents (10), and false hope (18, 19). False hope in fertility preservation occurs when patients believe that preserving their fertility will result in a future pregnancy and live birth, even though the chances of success are not so high or the desired outcome may not be achievable. This can happen when the limitations, uncertainties, and success rates of fertility preservation are not fully understood or clearly explained (20).

Among the most discussed ethical challenges of fertility preservation are the provision of equal access to these technologies, the age limits, and funding. There is a distinction between fertility preservation for medical and non-medical indications. Fertility preservation of user groups such as women opting for social egg freezing (21, 22) and transgender individuals (23) is often not covered by health insurance. Even within medical indications, the coverage varies by country, diagnosis, and patients’ age. Age limits generally apply to the assisted reproductive treatments required to use the preserved reproductive material. All countries apply upper age limits and some countries also have lower age limits for assisted reproduction (24). Some public health systems cover certain fertility preservation procedures, specifically for children, adolescents (25), and cancer patients, but this is not the case everywhere (26). The unequal access touches upon the issue of social justice.

Other ethical challenges are the participation of paediatric patients in the decision-making regarding their fertility preservation and the postmortem reproduction. Children's participation in medical decision-making is based on the ethical principle of respect for developing autonomy. While parents or legal guardians generally provide consent for medical treatment, children should be involved in decisions to an extent that reflects their age, maturity, cognitive development, and ability to understand the presented information. Rather than relying on a strict age threshold, contemporary ethical frameworks emphasise a child's decision-making capacity and encourage increasing participation as children mature (27).

Postmortem reproduction is the use of assisted reproductive technologies to achieve conception after the death of one genetic parent through the use of that individual's preserved gametes or embryos (28). Postmortem reproduction by a partner is forbidden in Germany, while it is allowed in some other countries and additional conditions are set: written consent by the deceased person, the partner received extensive counselling and a minimum waiting period of 1 year is imposed before a treatment can be started (28).

Against the background of more theoretical research in fertility preservation, our research provides real-life data on the opinions of professionals engaged in the everyday practice of fertility preservation. Our study aims at the identification and analysis of specific ethical challenges associated with fertility preservation through the perspective of experts in this field.

## 2 Methodology

In order to reach the aim, we investigated the perceptions of experts in fertility preservation. We have conducted a series of problem-centred, semi-structured exploratory interviews with 16 participants. Recruitment and interviews were conducted from March to July 2026. An invitation to participate was sent by email to 55 German experts. Only German experts were included for homogeneity of the studied group and an opportunity to ask questions regarding the health system access and funding of fertility preservation in the national context. The inclusion criteria were: clinical practice in fertility preservation, research activity on fertility preservation, German nationality. The experts were identified among the members of the professional networks FertiPROTEKT and Androprotect as well as through snowballing and publications on fertility preservation. FertiPROTEKT network is a German-speaking medical network focused on fertility preservation. It was founded in Germany in 2006 and brings together over 150 reproductive-medicine centers, hospitals, university clinics and fertility practices in Germany, Austria and Switzerland. FertiPROTEKT helps people preserve their ability to have biological children when a medical treatment might damage fertility. The network also develops medical recommendations, collects anonymised treatment data in a registry, and works on improving fertility-preservation techniques (29). Androprotect is a German medical network and clinical programme focused specifically on preserving male fertility, particularly in boys and young men whose fertility may be damaged by cancer treatment or other diseases (30).

Two reminders were sent out, and interested participants were asked for a suitable date and time of the interview. Additionally, written informed consent was obtained from the participants. Interviews were conducted via videoconference by S.A.Y. The interviews lasted 22 min on average and were recorded with the consent of the participants.

Semi-structured interviews are an established research method in applied medical ethics. It allows to gain insight into the subjective perspectives of the interviewees on the topic of research. Semi-structured interviews also allow flexibility in the conduct of the interview, and asking *ad hoc* questions for clarification of interviewees’ statements (31).

The interviews began with a brief presentation of the project. Participants had the opportunity to ask questions. The interviews were conducted with an originally developed interview guide which covered 5 topics: (i) autonomy and informed consent, (ii) beneficence, (iii) non-maleficence, (iv) social justice, (v) legal framework. The interview guide was developed on the basis of literature review and previous works of the authors’ team. Altogether, the interview guide consisted of 12 open-ended main questions and 5 follow-up questions. Digitally recorded interviews were transcribed and anonymised to protect participants’ privacy. A quality check was performed by listening to the audio files to check for any errors.

Thematic analysis was further performed by one member of the research team in accordance with Braun's and Clarke's latest recommendations for single coder (32). Thematic analysis proceeded according to sequential phases prescribed for this method: (i) familiarization with the data, (ii) generating initial codes; (iii) search for themes; (iv) reviewing themes; (v) defining and naming themes; (vi) producing the report (33). The results were discussed within the authors’ team. Our interdisciplinary team comprises an ethicist and public health expert (S.A.Y.), ethicist and political scientist (M.O.), physicians-gynaecologists (K.H. and K.B.), and physician and expert in ethics and history of medicine (F.S.). Inductive thematic analysis was applied. First, the inductive thematic analysis of the full text of the interviews was conducted. The coding process was performed manually and involved highlighting relevant ethical issues in the interviews. Then, the identified codes were clustered into themes and subthemes. Each theme was developed from multiple codes that identified different facets of the focus of the theme. The subthemes focused on one particular aspect of the theme (32). Recurring topics mentioned during the interviews were connected to representative quotes in order to illustrate the results.

## 3 Results

### 3.1 Participant characteristics

A total of 16 experts took part in the study. These were practicing physicians and researchers involved in fertility preservation. While all experts were involved in research on fertility preservation, one expert was only involved in research without being involved in clinical care of patients with fertility preservation. Five experts were men and eleven were women. The majority of experts had between 10 and more than 20 years of experience in fertility preservation - Table 1.

**Table 1:** Participants’ characteristics.

Characteristics	Number
Gender	
Female	11
Male	5
Years of experience	
< 10	4
10–20	6
20 >	6
Specialty	
Gynaecologists	6
Andrologists	3
Paediatricians	2
Oncologist	1
Psychosomatic medicine	1
Clinical embryologist	1
Biologist	1
Researcher	1

### 3.2 Themes

**Figure 1:**
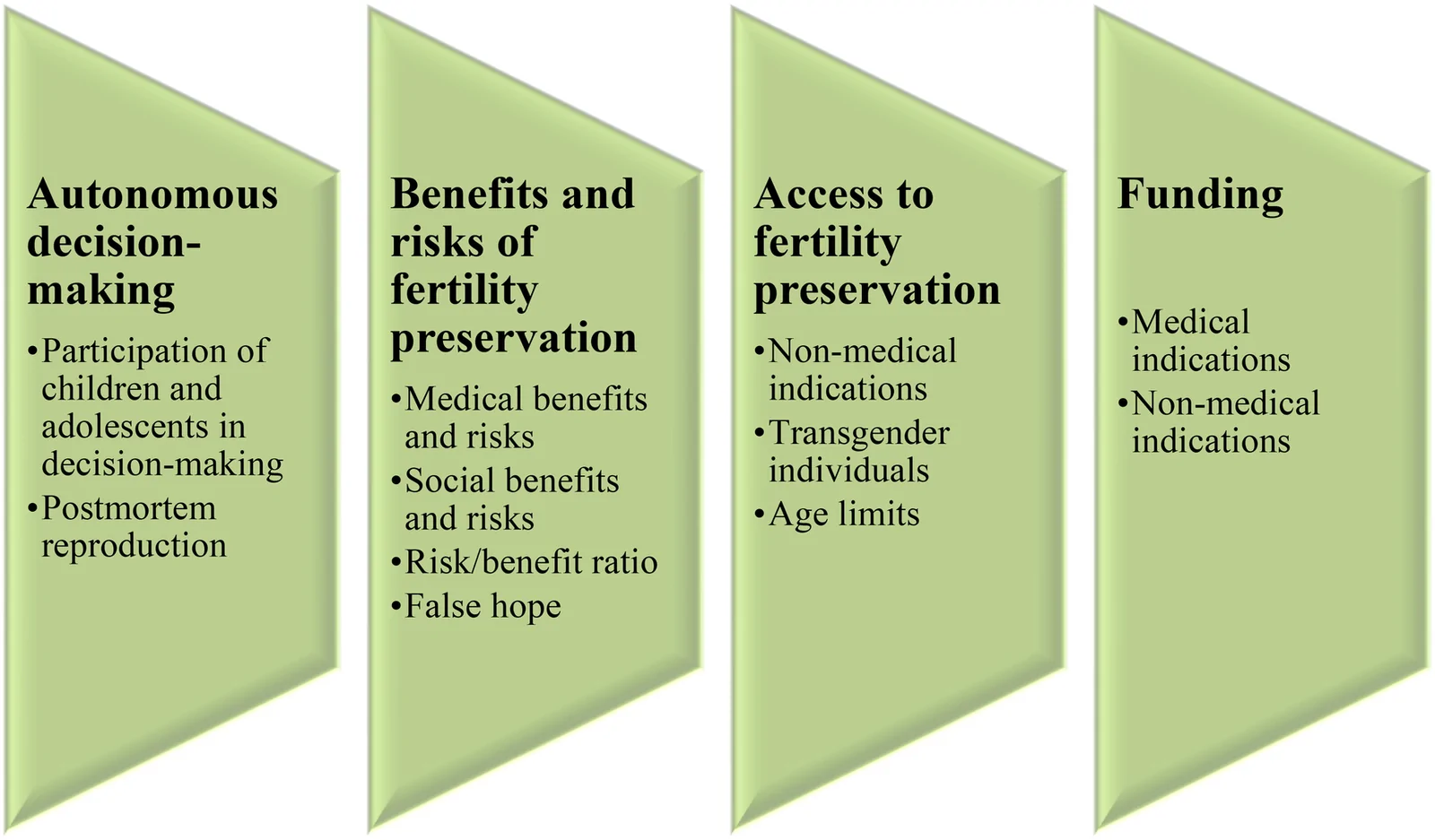
Themes and subthemes identified in the thematic analysis.

Thematic analysis identified four major themes: 1) autonomous decision-making; 2) benefits and risks of fertility preservation; 3) access to fertility preservation; and 4) funding. Within the themes, sub-themes were identified (Figure 1).

#### 3.2.1 Autonomous decision-making

##### 3.2.1.1 Participation of children and adolescents in decision-making

Most experts supported the involvement of paediatric patients in the decision process regarding their fertility preservation. Children's and adolescents’ perspectives might differ from the parents’ perspectives and they might ask intelligent questions (i5). Children should be involved depending on their stage of development and whether they are able to understand the whole situation (i2, i9, i13, i15). They should be involved “*to the same extent that children are typically involved in other medical decision-making processes concerning their own bodies.”* (i9). Experts also thought that the involvement of children should be decided in each individual case.

Further, experts considered that despite the involvement of paediatric patients in the decision-making, the final decision should be taken by the parents (i10, i13).

“I would always involve them [children], but I wouldn't let them make the decision entirely on their own.” (i10)

There was also an opinion opposing the involvement of children in the decision-making:

“I think that’s a tough issue for a child to grapple with. They [children] can’t understand it. It’s hard to understand what that means. It’s too much of a burden of responsibility for such a young child. I wouldn’t do it; I wouldn’t feel comfortable with it […] children should play and be loved, and do not have to think about fertility or anything like that, especially since they don’t understand it.” (i12)

Most experts pointed to puberty, i.e., around 14 years of age, as the age at which children should be involved in the decision-making. Already between 12 and 14 years, “*the children are strong enough to decide for themselves”* (i3) and “*it is the individual decision of the doctor whether the child is sensible enough”* (i3) to be involved in the decision-making. One expert pointed to 9 or 10 years of age “*when the body starts to change.”* (i11). Generally, children are involved if the physician thinks that “*the child has a certain level of maturity and the parents agree.”* (i6) The conversations are usually held in the presence of the parents to provide some support to the child (i16).

One expert was of the opinion that when children reach an age where they can understand what was done, they should be informed again to confirm whether they still want the frozen samples to be stored (i16).

Additionally, the experts recognised a need for research on the topic of the involvement of children in order to answer questions like “*How to involve children in a way that's appropriate for their age?”* and *“Whether it is better to leave the decision entirely to the parents?” (i11).*

Children can be enabled to understand what fertility preservation is and why it is important for their future. This can be achieved through sex education already at school (i1, i3) and by a gynecologist at initial examinations (i1) or in the specific situation of discussing fertility preservation (i5). The latter is not a stand-alone informational discussion, but it has to be integrated into the overall context (i16).

“Whatever information you wish to convey to the children should be presented in a way that is appropriate for their age and level of understanding.” (i8)

Playmobil figures (i13), illustrated brochures (i15), videos, and flyers (i2, i14) with age-appropriate pictures (i4) can be useful in these discussions.

##### 3.2.1.2 Postmortem reproduction

Currently, postmortem reproduction is forbidden in Germany. When asked whether postmortem reproduction should be allowed, the experts diverged in opinion. While some experts thought that postmortem reproduction “*under no circumstances should be allowed”* (i4, i5) and they “*would not do it, nor would allow it”* (i7), others thought that it should be possible (i2, i3) with the explicit consent of the person (i1, i2, i11, i13) and their partner (i16) so that “*the deceased's wishes are respected”* (i11).

“A legal framework would need to be established that would allow for this [postmortem reproduction], similar to a living will, so that the sperm can still be used after death.” (i11)

“I don't understand why it [postmortem reproduction] is prohibited. Because you also have the possibility in Germany to do a single mother treatment. You call it single mother by choice. And if this is allowed, I don't see any reason any longer to prohibit postmortem reproduction for a single woman with a husband who had died, maybe due to a disease or to other causes.” (i3)

One expert looked at the individual situation and thought that postmortem reproduction can take place “*in a relationship where death occurs at a time when family planning isn't yet complete but was already underway.”* (i16).

Most of the experts considered the discussion of postmortem reproduction necessary within the informed consent process, although they would not include it from the very beginning of the information process.

“So, you have to explain that [postmortem reproduction]. It’s not legally permitted in Germany. Of course, it’s possible to send the samples abroad and possibly do something with them there, but I don’t think that’s something you need to mention right at the very beginning. Maybe that needs to be explained as things progress.” (i7)

One expert’s opinion was against the discussion of postmortem reproduction:

“I feel that this falls into a category where, if the individual in question does not survive their illness, then the issue of fertility preservation itself is no longer relevant. So, I believe the answer is no.” (i10)

#### 3.2.2 Benefits and risks of fertility preservation

##### 3.2.2.1 Medical benefits and risks

Experts distinguished between reproductive and endocrinological benefits. The reproductive benefit concerned the ability to have one's own children. Endocrinological benefits concerned regaining the hormonal function when preserved ovarian tissue is transplanted (i3, i4).

“[…] an endocrinological benefit, because if you do fertility preservation with cryopreservation of ovarian tissue and you transplant the ovarian tissue after the treatment, then you have mostly an activation of the ovary and sometimes also an activation of the contralateral ovary. And you have maybe spontaneous cycles for three, four, five years.” (i3)

Additional benefits of the achieved higher estrogen levels are “*lower risks for osteoporosis, for cardiovascular disease, for mental disease, and also for sexual dysfunction.” (i3).*

One expert saw the process of fertility preservation in men as “*entirely positive*” with “*no inherent risks*.” (i8) Another expert saw only advantages of fertility preservation and thought that it is very safe (i11).

Other experts pointed to medical risks of fertility preservation, such as the risk of infection through testicular biopsy (i15), risks of laparoscopy, malignant cells in the transplant (i2, i6), risks to the quality of the sample (i16), injury to other organs, bleeding (i3, i5, i6, i11), and later pregnancy complications (i3). Fertility preservation postpones the main treatment (i3, i10, i14). Other risks are related to the lack of a guarantee of success (i5). There are also risks accompanying fertility treatment that the female partner needs to undergo (i15). There are no increased risks for future children (i16).

##### 3.2.2.2 Social benefits and risks

Fertility preservation allows to “*exercise the right to reproduction*”, to “*control reproductive options at any time”*, “*to reproduce regardless of physical condition or life stage”* (i1), and “*to preserve a lifelong dream”* (i10). Patients “*have a prospect”* and “*can shape their future”* (i4).

“It [fertility preservation] has one major benefit - namely, fulfilling the objective of the fundamental right to family planning and reproduction.” (i9)

In cancer patients, fertility preservation provides relief because the patient no longer has to worry about the effects of cancer treatment on fertility (i16). It also helps to protect the patient's relationship, if there is one (i16). Fertility preservation further helps in managing the main disease by creating hope and a positive psychological effect:

“But if we talk about cancer patients, for example, it has been shown that actually thinking about what it would mean to be able to start a family after successful treatment also has a very positive effect on the psychological side.” (i15)

Regarding induced risks, fertility preservation carries the risk of emotional distress (i13). It is also related to pressure on the patients. The pressure might be exerted by the reproductive partner, the family, or the employers who offer coverage of egg freezing (i1). Also, an important question is how the whole thing is secured financially, i.e., who bears the costs (i1). There is a likelihood that frozen material will not be used eventually (i5).

##### 3.2.2.3 Risk/benefit ratio

The experts considered that each case should be evaluated individually to determine whether the potential for genetic parenthood justifies the risks associated with fertility preservation (i1, i7, i13, i15).

“As is always the case in medicine, we have an indication, so to speak, and then we simply have to assess the risks and benefits involved, and this must also be weighed together with the person who is interested in it.” (i1)

“You always have to look at each case individually, of course. I don’t think you can apply a one-size-fits-all approach.” (i13)

Generally, the risks are low, i.e., 1%–2% (i3). The benefits are greater, which justifies the risks (i3, i12, i13).

Also, the risk/benefit ratio depends on the patients and their perspective. Some patients are willing to take the greatest possible risk (i4).

##### 3.2.2.4 False hope

Experts thought that to avoid the risk of false hope, a “*good explanation*” (i4), “*full and proper information*” (i1), “*patient-based and honest counseling*” (i3), and “*highly individualized consultation*” (i5) should be provided. The information should be provided both verbally and in writing because not all details may be understood by the patient during the consultation.

“We don’t make promises […] We don’t give any guarantees […] and not an exaggerated, false hope.” (i12)

“It may also be necessary to provide supplementary information in writing, simply because not all details may be fully absorbed by the patient during the actual consultation session. So, the point - the crucial point - is to repeatedly inform the patient, both verbally and in writing.” (i8)

One expert thought that physicians should provide an explanation based on “*the data published in the literature”* (i7). Another expert pointed to the provision of “*statistics on the success rates of artificial insemination.*” (i6)

#### 3.2.3 Access to fertility preservation

##### 3.2.3.1 Non-medical indications

Most experts were of the opinion that fertility preservation technologies should be accessible for non-medical reasons, such as social egg freezing.

“They [the women] should decide for themselves, so of course it [fertility preservation] should also be accessible to them so they can go through with it if they decide to do so.” (i6)

One expert emphasised the importance of ensuring that the system is not subject to misuse:

“In principle, yes, make it available, but also conduct a case-by-case review of the underlying circumstances and weigh all the risks […] So, don’t just make it available - take a closer look at exactly what it’s for and whether it really makes sense.” (i13)

One expert was of the opinion that women have long reproduction period, i.e., from 18 to 42 years, and if they really want to have children, then “*there is enough time to do it”* (i2). In that way, social egg freezing was seen as not needed.

As for the social sperm freezing, one interviewee believed that it should be accessible to everyone and “*a broad societal discussion should take place regarding when and how such measures should be undertaken.”* (i8).

##### 3.2.3.2 Transgender individuals

Most experts thought that transgender individuals should have access to fertility preservation technologies.

“I believe there is absolutely no reason to exclude certain groups of people from this [fertility preservation].” (i1)

“I wouldn’t rule them out just because they’re transgender [..] I do think transgender people should have access to it [fertility preservation].” (i13)

One expert was hesitant about the importance of fertility preservation to transgender individuals:

“I don’t really get the feeling that they [the transgender individuals] want that all [fertility preservation] that much […] Of course, you do everything you can for every patient group, but I don’t think that’s the main issue for them. So, I’m not sure if we should invest so much in that.” (i12)

##### 3.2.3.3 Age limits

The opinions of experts differed concerning age limits. Some supported an upper age limit, but not a lower age limit. Individual case-by-case decision was also supported:

“I would say there should be no low age limit and the upper age limit maybe should be in general around 40 years for women and 50 years for men. I think it is justified. But there should be also a possibility for the fertility specialist to make a decision on individual basis due to the reproductive potential of the individual patient.” (i3)

Some experts advocated for “*not setting any limits at all, neither for men nor for women*.” (i1).

“Purely on the basis of age, there shouldn't be any restrictions on access - specifically regarding an upper age limit.” (i9)

“I don’t think a rigid age limit makes sense. I mean, people are different, and biological age varies.” (i13)

#### 3.2.4 Funding

##### 3.2.4.1 Medical indications

All experts unanimously supported health insurance coverage of fertility preservation for medical indications. It was seen as a matter of social justice.

“[…] if the fertility decline is due to a disease, for example cancer disease, endometriosis, premature ovarian insufficiency, I think then it is an issue for the health insurance because it was not the choice of the patient.” (i3)

“[…] the risk or danger then [without health insurance] arises that only those with greater financial means will be able to afford it [fertility preservation], which is, naturally, quite unfortunate.” (i10)

One expert suggested copayments dependent on income and net worth:

“If there is a medical indication… I’d make that [the payment] dependent on - given that health insurance funds are becoming increasingly scarce - I’d make it partly dependent on income and net worth as well, and I’d favor a solution involving copayments for those with higher net worth. So, kind of like long-term care insurance.” (i13)

##### 3.2.4.2 Non-medical indications

Most experts didn't support health insurance coverage for social egg freezing. Social egg freezing was seen as a matter of personal choice, and as such, it should be paid by the individual and not by the health insurance.

“It’s essentially a personal choice, and I don’t think that needs to be covered by health insurance, specifically statutory health insurance.” (i1)

The experts also recognised that individual payment creates unequal access.

“Someone who wants to postpone parenthood for social reasons - because they want to focus on their career first - should pay for it themselves; they have the money for it. […] Then it gets complicated again, because then we have a medical system that favors only those who have money.” (i7)

On the other side, this payment was seen by one expert as a fact that regulates how the technology is being used, i.e., not lightheartedly but only by patients who really want and need fertility preservation.

“[…] in the past, of course, the fact that women had to pay for part or possibly all of the procedures themselves helped identify those who really, really wanted them. […] And if cost coverage weren’t an issue at all, I could imagine that some women who might not actually need it - because their ovarian reserve is very good or the chemotherapy isn’t as gonadotoxic - might use these procedures too lightly, even though they don’t really need them.” (i5)

There were experts who thought that the health insurance system is already overloaded and should not cover social egg freezing:

“I believe that if we were to make egg freezing readily accessible to everyone - even in the absence of medical indications - it would be a financial burden that the system simply could not sustain […] I tend to think that our efforts would be better directed toward providing extensive public education - to ensure that, in this day and age, the desire to have children isn't continually postponed until it is too late - rather than attempting to make egg freezing accessible to absolutely everyone. I simply don't believe that is something our society can realistically afford to do.” (i10)

Another situation classified as non-medical by some of the experts was fertility preservation in transgender individuals. Being transgender was clearly considered by one expert as a non-medical condition, “*a matter of identity*”, and as such, it shouldn't be covered by the health insurance (i1). Another expert equated fertility preservation in transgender individuals to social freezing and as such “*it shouldn't be a routine benefit covered by health insurance*.” (i11).

“If I’m a healthy man or a healthy woman, I can’t expect a healthcare system that’s already stretched to its limits to routinely cover these costs for me when they aren’t covered for people who aren’t transgender.” (i11)

One expert supported a case-by-case review based on criteria and performed by a panel of experts against automatic health insurance coverage (i13).

Other experts supported health insurance coverage for fertility preservation in transgender individuals:

“[…] if they [health insurance companies] finance the operation […] if they finance the hormone therapy, I think then they can also finance the cryopreservation of sperm or oocytes [of transgender individuals]. And I think then it should be covered by health insurance.” (i3)

## 4 Discussion

We analysed our findings through the four principles of bioethics. The principle of respect for autonomy requires the involvement of the patient in decision-making and the obtaining of informed consent. Fertility preservation *decision-making* involves children and adolescents in case of cancer diagnosis as well as gender reassignment treatment. Although the main focus is on cancer treatment, respectively gender dysphoria treatment, decisions about fertility preservation are also important because they may have significant implications later in adulthood (34). Our interviewees supported the involvement of children and adolescents in decision-making, provided that the child is mature enough. In general, children under the age of 18 cannot give consent for medical procedures and treatments. Parents are typically the primary decision-makers for their children. However, in paediatrics, children who are grown up enough to understand medical discussion are asked to give assent for care (35). The age at which assent is required varies. Our interviewees thought that children around 12–14 years can already be involved in the decision-making, which is in accordance with existing guidelines (36). Even younger children can be involved with age-appropriate information provision (37) and a case-by-case assessment of children's competence (36).

Research on fertility preservation in paediatric and adolescent oncology shows that children and adolescents want to be involved in decisions about their future fertility, although parents and clinicians often retain primary control. It was found that adolescents frequently express concerns about future parenthood and prefer shared decision-making rather than exclusion from discussions (34). Recent studies show that adolescents and young adults value fertility counselling highly and report greater satisfaction with care when fertility risks and preservation options are discussed early and comprehensively (38, 39). Additionally, adolescents generally report greater satisfaction when they receive age-appropriate information and are included in conversations about risks, options, and future reproductive goals. Exclusion from decision-making, on the other side, may contribute to later regret and reduced trust in healthcare providers (40). The ethical importance of assent, autonomy, and family-centred counselling is emphasised. Healthcare professionals are encouraged to involve children according to developmental capacity, balancing respect for emerging autonomy with parental responsibility and medical urgency (41).

Another aspect that we analysed in relation to autonomous decision-making was the inclusion of *postmortem reproduction information* within the informed consent for fertility preservation. The experts supported information provision, but some of them opposed to the allowance of postmortem reproduction itself. In countries where postmortem reproduction is permitted, discussion of postmortem use during informed consent for fertility preservation is essential because explicit prior consent of the deceased is required for authorising postmortem reproduction (24, 42). It also represents respect for the autonomy of the deceased. In countries where postmortem reproduction is forbidden, like Germany, the discussion is still necessary because patients have the right to know the fate of their preserved reproductive biomaterial in case of death. Although the experts did not discuss postmortem reproduction in depth, it is an issue intertwining the autonomous decision of the deceased, the rights of the partner to choose with whom to reproduce, and the interests of the future child who will be raised by only one parent.

The principles of beneficence and non-maleficence require identification of *benefits and risks of fertility preservation*. They have been widely discussed in the literature (43–45). Our interview partners were focused mainly on the medical benefits and risks, which might be due to the fact that they were practicing physicians. The risk/benefit ratio of fertility preservation was in favour of the benefits, but needs to be assessed in each case individually and with the participation of the patient. Among the risks, special attention was paid to the risk of false hope as it can be reduced with proper informing.

Fertility preservation offers important medical, psychological, and social benefits for individuals at risk of infertility due to cancer treatment, other medical conditions or age-related fertility decline. The primary benefit is the possibility of achieving biological parenthood in the future, fulfilling the objective of the fundamental right to family planning and reproduction. This may improve long-term quality of life for all patients undergoing fertility preservation. As for fertility preservation for medical indications, it reduces regret associated with treatment-related infertility. Additionally, fertility preservation may provide emotional reassurance and hope during a period of significant uncertainty for cancer patients (38, 39).

Fertility preservation also involves risks. In healthy women opting for social egg freezing most common is the risk of decisional pressure on the side of employers covering the expenditure for the fertility preservation or future partner's expectations (17). In cancer patients, pressure might come from the physicians trying to avoid delay in anti-cancer treatment. Further, many procedures are invasive, expensive, and associated with uncertain success rates. In female patients, ovarian stimulation and oocyte retrieval may delay cancer treatment and carry risks related to hormonal exposure or surgery. Ovarian tissue cryopreservation in prepubertal girls shows promising results, but the experience is still limited. As for testicular tissue cryopreservation, it is still considered experimental and has uncertain long-term efficacy that raises ethical concerns regarding future use and consent (46, 47). Decision-making often occurs shortly after diagnosis under considerable emotional stress, which may impair understanding and contribute to decisional conflict or later regret. Recent reviews emphasise that unmet informational and emotional needs, insufficient counselling, and lack of developmentally appropriate communication are strongly associated with decision regret among adolescent and young adult cancer survivors (48). Thus, communication with patients takes a priority place in fertility preservation care. This is also the case when the communication should help to avoid false hope. Our interviewees underlined the importance of full, proper and individually tailored information provision both orally and in written form.

The principle of social justice supports equal treatment of different groups of patients. In our study, experts expressed their opinion about the *access of different patients’ groups* to fertility preservation and funding.

Some experts supported an upper *age limit* for access to fertility preservation technologies. It was related to the biological natural limits of human reproduction, i.e., around 40 years of age for women and 50 years of age for men. Other experts advocated for setting no limits or a case-by-case approach.

Age is one of the most important determinants for success in fertility preservation. Scientific literature shows that ovarian reserve and oocyte quality decline with advancing age, which directly affects the outcomes of cryopreservation and future pregnancy rates (43, 49). Different fertility preservation techniques therefore, have different practical and recommended age limits. For women undergoing oocyte or embryo cryopreservation, guidelines emphasize that younger age at the time of preservation is associated with significantly better reproductive outcomes. The European Society of Human Reproduction and Embryology (ESHRE) guideline on female fertility preservation states that oocyte cryopreservation is the preferred method for postpubertal women, but success strongly depends on the woman's age and the number of oocytes stored (43). In ovarian tissue cryopreservation age is particularly important because the ovarian follicle pool decreases progressively over time. Several studies identify approximately 35 years of age as a practical upper threshold for ovarian tissue cryopreservation due to declining follicular density and reduced transplantation success. A review on ovarian tissue cryopreservation in children and adolescents reported that “35 years of age is considered as a limit for cryopreservation techniques” because primordial follicle numbers decrease substantially after this age (50). However, recent evidence suggests that strict upper age limits may need reconsideration. A study of Patel et al. reported successful pregnancies and live births in women older than the traditionally accepted age limit for ovarian tissue cryopreservation, indicating that individualized assessment may be more appropriate than rigid chronological cut-offs (51). Such an approach is also more ethical, respecting the equity and participation of patients in decision-making. For paediatric patients, fertility preservation can be offered before puberty. Thus, fixing lower age limits is also ethically inappropriate. Ovarian tissue cryopreservation is currently the only available fertility preservation option for prepubertal girls facing gonadotoxic treatment. Studies demonstrate that the procedure is feasible and increasingly used in paediatric oncology and hematology settings (52). Professional societies such as the American Society for Reproductive Medicine and ESHRE do not define absolute universal age cut-offs for fertility preservation. Instead, they recommend individualized counseling based on ovarian reserve, diagnosis, urgency of treatment, and expected reproductive potential. Nevertheless, outcomes decline significantly after the late 30s and especially after age 40 (43, 53).

Our interviewees supported *access for non-medical indications*, i.e., social egg freezing, but they thought that these patients should pay for fertility preservation alone and not through the health insurance, which *per se* creates unequal access. Some experts expressed concerns that health insurance coverage of fertility preservation for non-medical indications can burden the healthcare system enormously. Decisive consideration was whether patient's situation was a matter of choice. Thus, social egg freezing should not be covered by health insurance as it is seen as a matter of personal choice. Unresolved remains the question of social justice because not all patients can afford the expensive fertility preservation procedures when they need to pay for them out of pocket. On the other side, there is the example of France where social egg freezing is covered by health insurance (54). In the long run, it is possible that more countries will apply such funding. It is also questionable to what extent women opting for social egg freezing are free and well informed in their decision-making, bearing in mind that the procedure is offered mainly by private clinics that may not provide extensive information about their success rates and procedure-related risks (55). However, we have to point out that undue pressure may also occur in medically indicated fertility preservation. For example, children and adolescent cancer patients may experience pressure on the side of their parents (56).

Another group that should have access to fertility preservation technologies, on the opinion of our experts, was the group of *transgender individuals*. Being transgender was seen by most of the experts as a medical condition and fertility preservation in that case should be covered by health insurance. In Europe, there is a variety of approaches to transgender individuals regarding fertility preservation (24). In 25 European countries cryopreservation of reproductive material of transgender individuals was allowed (24). Still, the range of services offered to transgender individuals remained narrower than for cisgender patients (24). In Germany, public statutory health insurance typically covers fertility preservation when it is required due to potentially fertility-damaging medical treatments like chemotherapy, radiotherapy involving the reproductive organs, surgical removal of gonadal tissue, or other therapies with a substantial risk of fertility impairment. Fertility preservation for transgender individuals is a recognised medical right. Statutory health insurance is legally mandated to cover the costs of cryopreservation (freezing of sperm or eggs) and associated medical measures before gender-affirming treatments that risk future infertility. The extraction, cryopreservation, and storage costs are covered. However, the actual use of these preserved cells (such as *in-vitro* fertilization or other forms of artificial insemination) is subject to different legal guidelines and may only be partially covered or fully out-of-pocket, depending on the patient's marital status and specific insurance plan (57). Besides the cost of treatment, other barriers to fertility preservation access in transgender individuals include: inconsistencies in form and timing of counseling, potential worsening of gender dysphoria with fertility preservation treatment, limited research on fertility preservation outcomes, and legal barriers (8).

All experts in our study supported *health insurance coverage* in case of medical indications for fertility preservation. Comprehensive insurance coverage for medically indicated fertility preservation should include specialist consultation, laboratory testing, ovarian stimulation medications, surgical retrieval procedures, cryopreservation, and storage fees for a medically appropriate duration. Coverage should also extend to paediatric and adolescent patients facing treatments associated with future infertility risk (53). ESHRE emphasizes that fertility preservation should be considered part of comprehensive patient-centered care for individuals facing infertility risks associated with medical treatment (43). ESHRE recommends that fertility preservation counseling and referral be integrated into standard oncological and reproductive care pathways (43). Despite these recommendations, substantial disparities in access to fertility preservation services persist among European countries due to differences in national health insurance systems and reimbursement policies (58). These create inequities in reproductive healthcare access across Europe.

A major development in Germany was the introduction of statutory health insurance coverage for fertility preservation measures associated with fertility-threatening medical treatments. Since the enactment of the law “Terminservice- und Versorgungsgesetz” in 2019, individuals insured under the statutory health insurance system have been entitled to reimbursement for the cryopreservation of gametes (oocytes and sperm) or gonadal tissue when a disease and its treatment are likely to cause infertility. The legal basis for this entitlement is provided by § 27a(4) of the German Social Code Book V (59). The practical implementation of this benefit was specified by the Cryopreservation Guideline.

Recent evidence suggests that, despite the expansion of insurance coverage, some patients continue to experience financial burdens associated with fertility preservation (60). Administrative requirements, variations in implementation, or gaps in coverage for certain clinical situations may still lead to out-of-pocket expenditures. Nevertheless, the German reimbursement framework represents a substantial improvement compared with the situation before 2019, when most fertility preservation procedures had to be self-funded (60). Germany has established one of the more comprehensive public funding systems for medically indicated fertility preservation in Europe. By integrating cryopreservation services into statutory health insurance for patients facing fertility-threatening treatments, policymakers have strengthened reproductive autonomy and improved long-term quality of life for individuals affected by serious diseases. Still debated are the topics of access, equity, and the exclusion of elective fertility preservation from health insurance reimbursement.

## 5 Limitations

Our research is limited by several conditions. Firstly, the qualitative methodology does not allow for representativeness of opinions. Secondly, to obtain a full picture of fertility preservation experiences, patients’ and their families' perspectives should be added. This is the goal of the next stage of our research. Thirdly, the inclusion of only German experts did not allow generalisation of results and international comparisons. Also, the fact that the participants were exclusively medical experts professionally involved in fertility preservation, results in a predominant clinical perspective and limit the diversity of presented views. Nevertheless, we were able to obtain rich perspectives from the experts on their perceptions of fertility preservation and to fulfil our research aim.

## 6 Conclusions

The results highlighted the variety of experts’ views on fertility preservation. The decisions about fertility preservation are decisions influencing the rest of the life of the patients and the fulfilment of the right to reproduction. According to the opinions of the experts in the study, all patient groups that can benefit from fertility preservation technologies should have access to them, including minors, healthy women, and transgender individuals. This could be achieved through the coverage of fertility preservation by health insurance. Germany is among the European countries with the most comprehensive public funding schemes for medically indicated fertility preservation. In the long run, fertility preservation for non-medical indications could also be covered by health insurance, as this has been done in France. The experts underlined the importance of informed consent, particularly in paediatric cancer cases, where the involvement of children in decision-making is supported in accordance with their maturity. Another ethical challenge was the postmortem reproduction, on which experts’ opinions diverged. Wider public debate on its legal allowance might be beneficial. In any case, patients should receive information about postmortem reproduction to respect their right to know what will happen to their preserved reproductive biomaterial in case of death. As successful as fertility preservation technologies might be, full, proper, and individualised information should be provided to patients to avoid the risk of false hope.

## Data Availability

The original contributions presented in the study are included in the article/Supplementary Material, further inquiries can be directed to the corresponding author.
