# Supplementary material for: Ethical perspectives of fertility preservation through experts’ opinions
Source: Front Reprod Health. 2026 Sep 8;8:1952541. doi: 10.3389/frph.2026.1952541 (PMC13593903; doi:10.3389/frph.2026.1952541)
Supplement: Supplementary file 1 [file Table1.docx]

Supplementary Material

| **INTERVIEW GUIDE** | |
| --- | --- |
| **Topics and questions** | **Content-related focus** |
| **Project Information**  The FePro project involves research on fertility preservation in women, men, children, and transgender individuals from molecular, pathophysiological, genetic, ethical, and psychosomatic aspects. The aim of the FePro project is to investigate the needs of these patients and their partners/family and consequently, to develop preventive and helpful interventions.  The aim of the ethical sub-project within FePro project, in which you participate, is to provide systematic and in-depth knowledge of the ethical, social, and normative challenges associated with fertility preservation.  For fulfilling this task, we use a qualitative research approach interview. The interview will last approximately 30 minutes. The interview will be digitally recorded, stored on Ulm University’s secure servers, and then transcribed. All data will be anonymised and treated confidentially. Only the researchers in this team will have access to the recordings and transcripts.  The results will be published in scientific articles and publicly presented at scientific events in fully anonymised form. | **Research subjects and studied aspects**  **Aim of the ethical sub-project**  **Interview procedure**  **Data processing**  **Results utilisation** |
| **Introduction to the questions**  In our interview, we would like to ask you about your fertility preservation experience through the lens of your specific area of expertise. We would also like to investigate your concerns regarding the usage of fertility preservation technologies and your view about possible improvements. | **General questions’ content** |

| **Questions** | **Follow-up questions** | **Content-related focus** |
| --- | --- | --- |
| **Introductory questions** | | |
| 1. Do you consider research on fertility preservation as important and timely? |  | **General attitude to fertility preservation research** |
| 2. For how long have you been involved with questions related to fertility preservation? |  | **Experience with fertility preservation** |
| **Topic: Autonomy and informed consent** | | |
| 3. Should children be involved in the decision process regarding fertility preservation? | - *At what age should children be involved in this decision?* - *How can children be enabled to understand what fertility preservation is and why it is important for their future?* | **Involvement in research of people who are not autonomous to decide for themselves** |
| 4. Do you think that postmortem reproduction should be discussed as part of the informed consent for fertility preservation? |  | **Information content** |
| **Topic: Beneficence** | | |
| 5. What benefits of fertility preservation do you see? |  | **General benefits** |
| **Topic: Non-maleficence** | | |
| 6. What risks of fertility preservation do you see? |  | **General risks** |
| 7. In your view, what should be done to avoid the risk of false hope related to fertility preservation? |  | **False hope** |
| 8. Do you think that the chance of genetic parenthood justifies any potential risks of fertility preservation? |  | **Risks-Benefits ratio** |
| **Topic: Social justice** | | |
| 9. Do you think that fertility preservation should be covered through health insurance for medical and non-medical indications? | - *Do you think that fertility preservation technologies should be accessible for non-medical reasons, e.g., social egg freezing?* | **Health insurance coverage** |
| 10. Do you think that transgender individuals should have access to fertility preservation technologies? | - *Do you think that fertility preservation should be covered through health insurance for transgender individuals?* | **Transgender individuals** |
| **Topic: Legal framework** | | |
| 11. Do you think that there should be age limits for access to fertility preservation technologies? |  | **Age limits** |
| 12. Do you think that postmortem reproduction should be allowed? | - *If yes, for which specific cases?* | **Postmortem reproduction** |
| **Closing remarks and information**  Thank you for your participation in this interview. Is there anything else that you would like to add? | |  |
